# Consultant Psychiatrists as Chief Executive Officers (CEOs): Competencies and Challenges

**DOI:** 10.7759/cureus.96286

**Published:** 2025-11-07

**Authors:** Abby Fayen, Alexander Mayor, M Aamer Sarfraz

**Affiliations:** 1 Psychiatry, Oxleas NHS Foundation Trust, London, GBR; 2 Psychiatry, Institute of Medical Studies, Kent, GBR

**Keywords:** ceo, education, leadership, medical leadership, psychiatrist

## Abstract

Background

Effective medical leadership is critical to delivering high-quality healthcare, with evidence linking strong leadership to improved patient outcomes. This study explores the insights and experiences of consultant psychiatrists currently working as Chief Executive Officers (CEOs) of mental health organizations within the United Kingdom (UK), examining how their clinical training influenced their leadership capabilities.

Methods

This study is a qualitative study with brief closed-item descriptors. The study population was consultant psychiatrists in the United Kingdom serving as CEOs of mental health organizations, a census of n = 5, with n = 4 responses to the survey. A questionnaire was designed by the authors for the purpose of this study. The questionnaire consisted of five multiple-choice questions and five open-ended questions. The results were analysed using descriptive statistics and Braun and Clarke’s thematic analysis.

Results

Of the four psychiatrist CEOs who took part in the study, all had over 20 years of psychiatric experience. Their duration of time as CEO varied, with 2/4 serving for one to three years, 1/4 for four to seven years, and 1/4 for over seven years. Of the aspects of psychiatric training most beneficial to the role of CEO, 4/4 felt that patient-centred care and analytical thinking were beneficial, and 3/4 felt that ethical decision-making, crisis management, empathy, and communication skills were beneficial. The participants highlighted clinical leadership and a deep understanding of human behaviour as major advantages of their medical background. However, challenges such as managing staff expectations, balancing clinical and administrative duties, time constraints, and emotional burnout were also reported.

Conclusion

Consultant psychiatrists possess transferable skills that align well with leadership roles, including CEO positions. Limitations of this study include the small sample size and the risk of identifiability of the consultant psychiatrist CEOs. This study did not require ethical approval as confirmed by the National Health Service (NHS)/Health Research Authority (HRA) decision tool. Participation in the study was voluntary, and responses were anonymised. While not all psychiatrists may aspire to such roles, targeted training and opportunities should be provided to support those interested in pursuing executive leadership in healthcare.

## Introduction

Leadership is defined as an individual’s ability to guide or influence a group [1]. In healthcare, medical leadership refers to physicians who assume management responsibilities, whether or not they maintain clinical roles [2]. Effective medical leadership is critical to delivering high-quality care, yet limited research compares the performance of hospitals led by medical versus non-medical Chief Executive Officers (CEOs) [3,4]. While physicians commonly hold leadership positions such as clinical directors, only a few ascend to CEO roles [5]. Perry and Mason suggest that medical leadership correlates with improved patient outcomes [6], and Clay-Williams et al. show that it underscores the value of physician representation on organisational boards [7].

In 2009, Goodall examined top United States (US) hospitals and found a significant positive association between hospital quality rankings and physician CEOs, particularly in cardiac, cancer, and digestive disorder care [8]. However, physicians transitioning to leadership roles require not only clinical expertise but also proficiency in healthcare management, necessitating targeted training in business acumen, leadership development, and cross-professional collaboration [3,9].

Internationally, Denmark and the US (notably Kaiser Permanente) offer instructive models for physician leadership. Denmark emphasizes structured training and support for doctors in leadership roles, while Kaiser Permanente demonstrates the impact of high physician representation in executive positions [5].

In the United Kingdom (UK), the National Health Service (NHS) Next Stage Review (2008) highlighted the need to integrate leadership into clinician training, advocating for investments in clinical and board leadership programs to equip doctors with skills for system-wide influence [10]. This vision was operationalised through the Medical Leadership Competency Framework (2010), which outlines core competencies for physicians, including strategic direction, personal qualities, teamwork, and service improvement [11]. The General Medical Council (GMC) further mandates leadership as a professional requirement, embedding it into foundational and specialty training curricula [12,13].

Psychiatrists possess transferable skills for leadership, such as conflict resolution, group dynamics, and rapport-building, that align with organisational governance [6]. Despite this, few psychiatrists hold CEO roles in UK healthcare [6]. Bhugra [14] argues that psychiatrist-led leadership is essential to addressing 21st-century mental health challenges, as psychiatrists can uniquely inspire, motivate, and empower teams.

Our study aims to explore the experiences and insights of consultant psychiatrists who have served as CEOs of mental health organisations, evaluating how their clinical training informs their leadership approach, in particular which competencies psychiatrist-CEOs perceive as most transferable and the main challenges in balancing the psychiatrist-CEO's role.

## Materials and methods

All consultant psychiatrists currently working in the UK as CEOs of mental health organisations were identified through professional networks. The consultant psychiatrists were not known by the authors personally. This identified n = 5 participants eligible to take part in the study. All five were contacted via email, explaining the purpose of the study. Participation was voluntary, with anonymity guaranteed. All five were sent a link to complete the questionnaire via email. The response rate to the questionnaire was n = 4. One participant did not choose to complete the questionnaire; the reason they chose not to complete the questionnaire was not disclosed. A questionnaire was sent to the participants instead of completing an interview in order to facilitate and encourage participation and to facilitate anonymity of responses to encourage honest feedback and minimise potential response bias. While with self-reporting there is a risk of bias, this cohort represented an expert review with feedback analysed with a high reliance on face validity. 

Due to the limited number of consultant psychiatrists currently working as CEOs in the UK, there is an identifiability risk. This risk has been attempted to be reduced by not specifying the area of the UK where the participants work and not including other identifiable details. The participants were educated on the purpose of this questionnaire prior to participation, and implied consent was taken from the participants through their decision to complete the questionnaire.

Prior to the development of the questionnaire, a literature review was conducted to examine medical leadership internationally, in particular Denmark and the US (notably Kaiser Permanente). Particular emphasis was placed on psychiatrists in the role of CEO. While this showed that there are many healthcare professionals in leadership roles within the healthcare system, it showed a minority in the role of CEO. The authors then also examined the UK training programme for doctors, both as a foundation doctor and a psychiatry trainee, and in particular, the leadership training emphasis within these training programmes. A pilot of the questionnaire was completed with two of the five consultant psychiatrist CEOs in order to improve and finalise the questionnaire. 

The structured questionnaire was developed based on the authors' understanding of the UK mental health system to systematically evaluate the relationship between psychiatric expertise and leadership effectiveness in CEO roles. The questionnaire was designed on SurveyMonkey and sent as a link via email to the participants. Response to the questionnaire was anonymous, including from the authors, removing the need to de-identify participants. 

The questionnaire was sent to and completed by all participants in October 2024. After the email containing the questionnaire was sent, two reminders were sent requesting completion of the questionnaire. There were no follow-up questionnaires sent, and no interviews took place. 

The questionnaire employed a mixed-methods design comprising two distinct sections. The five multiple-choice items examined demographic and professional characteristics, including duration of clinical practice and tenure in executive leadership. The questionnaire, as shown in the Appendix, shows the answers that the participants can choose from to answer the multiple-choice questions; all of these options were tick-box responses with the participant choosing to tick the box/boxes relevant to them. 

The qualitative section included five open-ended questions designed to elicit perspectives on how the participants' psychiatric training influenced their leadership philosophy, decision-making processes, and organisational management approach. 

All authors analysed the data. The data were analysed using Microsoft Excel and Microsoft Word (Microsoft Corp., USA). Data collection and analysis procedures adhered to the current General Data Protection Regulation (GDPR) regulations. Key thematic domains identified include the transferability of clinical skills to executive functions, unique challenges of dual clinical-administrative roles, and perceived advantages of medical training in healthcare leadership. Participants cited “understanding people and their behaviour” and “clinical leadership” as advantages of their background in psychiatry, and advices for aspiring consultant psychiatrists included “aim to maintain core identity and skill set”, “maintain relationships”, “be brave and make a difference”, and “find a mentor”. The participants did not provide feedback on the findings. 

Data from the multiple-choice questions was analysed using descriptive statistics to identify patterns in professional backgrounds and role durations. Qualitative responses underwent thematic analysis following Braun and Clarke's six-phase framework [15] to identify, analyse, and report recurring patterns of meaning. This approach allowed for both numerical trends and narrative insights to provide an understanding of participants' experiences. The reporting guideline, Consolidated Criteria for Reporting Qualitative Studies (COREQ) (32-item checklist), was used to analyze the questionnaire [16]. 

The NHS Health Research Authority decision tool identified this study as research [17] and also confirmed that this study does not require ethical approval [18].

## Results

In October 2024, a questionnaire was distributed to all psychiatrists in the UK serving as CEOs of mental health organisations (n = 5); it yielded an 80% response rate (n = 4). Table 1 shows the participant characteristics, with all respondents (4/4) having over 20 years of psychiatric experience. Their tenure as CEOs was varied: 2/4 had served for one to three years, 1/4 for four to seven years, and 1/4 for over seven years.

**Table 1:** Participant characteristics.

Characteristic	Participants (n = 4)
Years practicing as a psychiatrist	
11-20 years	0
More than 20 years	4
Years served as a CEO of the mental health trust	
Less than 1 year	0
1-3 years	2
4-7 years	1
More than 7 years	1

When asked which aspects of psychiatric training most benefited their CEO role, all respondents (100%) emphasised patient-centred care and analytical thinking. Additionally, 75% (n = 3) cited ethical decision-making, crisis management, empathy, and communication skills as key contributions to their leadership effectiveness, as shown in Table 2.

**Table 2:** Multiple-choice question response to "aspects of psychiatric training most beneficial for the role of CEO".

Aspects of psychiatric training most beneficial for the role of CEO	Participants (n = 4)
Patient-centred care	100% (4/4)
Analytical thinking	100% (4/4)
Ethical decision-making	75% (3/4)
Crisis management	75% (3/4)
Empathy	75% (3/4)
Communication skills	75% (3/4)

When describing how their psychiatric background influenced CEO-level decisions, recurring themes included interpreting group dynamics and applying clinical knowledge, with participants citing those themes. This highlights the beneficial nature of psychiatry training, both in developing working professional relationships and having an improved understanding of the health care environment in which you have a leading role. 

Regarding challenges in balancing clinical and executive duties, all responders (100%) identified maintaining clinical skills and managing staff expectations as their primary difficulties, as shown in Table 3. Administrative burden and time management were each noted by 50% (n = 2), while 25% (n = 1) reported emotional burnout. None selected role conflict as a challenge.

**Table 3:** Multiple-choice question response to "main challenges in balancing responsibilities as CEO".

Main challenges in balancing responsibilities as the CEO	Participants (n = 4)
Maintaining clinical skills	100% (4/4)
Managing staff expectations	100% (4/4)
Administrative burden	50% (2/4)
Time management	50% (2/4)
Emotional burnout	25% (1/4)
Role conflict	0% (0/4)

When asked "what advantages they have as a CEO because of your background in psychiatry", recurrent themes included deeper behavioural insight and strengths in clinical leadership, with participants citing “understanding people and their behaviour” and “clinical leadership”. This emphasises the importance of leadership experience for trainee psychiatrists in order to develop these skills. Psychiatry training also facilitates the development of good communication skills and an in-depth understanding of human behaviour. 

When asked about the "disadvantages of dual roles" themes centred on sustaining clinical competencies if/when they return to their clinical role full-time, with participants citing “difficulty maintaining clinical skills”. This reflection highlights the participants' recognition of their limitations due to the change in their clinical role.

When asked for "advice for aspiring consultant psychiatrists", common themes included preserving professional identity, fostering relationships, seeking mentorship, and demonstrating courage to drive change. Comments from participants include “aim to maintain core identity and skill set”, “maintain relationships”, “be brave and make a difference”, and “find a mentor”. Their reflections highlighted the broader impact on population health, the value of peer support, and meaningful contributions throughout the leadership journey.

All respondents encouraged psychiatrists to pursue leadership roles, citing “unique skill alignment”, as shown in Table 4.

**Table 4:** Multiple-choice question response to "Would you recommend other psychiatrists to pursue leadership roles such as CEO?"

Would you recommend other psychiatrists to pursue leadership roles such as a CEO?	Participants (n = 4)
Strongly recommend	100% (4/4)
Recommend	0% (0/4)
Neutral	0% (0/4)
Do not recommend	0% (0/4)
Strongly do not recommend	0% (0/4)

When asked if there was "anything else they would like to share about your experience as a psychiatrist and CEO?", comments from participants included “make a difference at every stage of leadership journey”, “have a greater impact on population health”, and “lean on peers for support”. These reflections emphasise the positive leadership experience that the consultant psychiatrists have had in their role as CEOs of mental health organisations. 

## Discussion

The findings of this study are consistent with a growing body of literature that underlines the distinct and valuable contributions of psychiatrists in leadership roles. The Royal College of Psychiatrists has emphasized that consultant psychiatrists possess a unique skill-set, positioning them to lead multidisciplinary teams in ways that not only optimise patient outcomes but also drive sustained organisational improvement [19]. In a similar vein, Bohmer [20] suggests that clinician-leaders function as essential advocates for patient welfare within complex healthcare ecosystems. The experiential narratives of leaders, such as Dr. Matthew Patrick, a psychiatrist-CEO, further reinforce this perspective. His insights illustrate how core elements of psychiatric training, particularly its emphasis on reflective practice and relationship-oriented approaches, are readily transferable to effective organisational leadership [6].

This study identifies a number of psychiatric competencies that participants perceived as directly contributing to their effectiveness as CEOs. These included patient-centred care, advanced analytical reasoning, ethical decision-making, crisis management, and the ability to foster psychological safety. Such competencies have echoed in the findings of Berghout et al. [1], who describe multidisciplinary collaboration, peer empowerment, and consensus-building as foundational tools for medical leadership. Participants in our research reported a capacity to interpret complex group dynamics and integrate clinical insight into executive-level decision-making. These capabilities align with Perry and Mason’s [6] contention that psychiatric training inherently fosters self-awareness, emotional intelligence, and nuanced interpersonal regulation; traits that are increasingly recognised as critical for successful leadership.

The leadership attributes described by our participants, such as understanding human behaviour and modelling clinical integrity, closely match those identified by Kahn et al. [1]. Nonetheless, our study also brought to light several substantive challenges encountered by psychiatrist-CEOs. These include navigating tedious administrative responsibilities, managing divergent staff expectations, and confronting risks of emotional fatigue and burnout. These challenges mirror broader international findings, which cite high CEO turnover rates in healthcare systems as a consequence of chronic stress, operational pressures, and inadequate structural support for executive leaders [1].

To address barriers, participants strongly advocated for enhanced access to mentorship and structured peer support networks. This recommendation is consistent with solutions proposed by Kahn et al [1] and supports the growing call for personalised leadership development initiatives tailored specifically for clinicians, particularly psychiatrists, transitioning into executive roles. Structured programs may help mitigate role-related stress and improve leadership sustainability by fostering resilience, strategic acumen, and a supportive professional community.

Strengths of this study include the census approach taken, where all consultant psychiatrists working as CEO’s within the UK were invited to take part in the questionnaire, with an 80% (n = 4/5) response rate. Their participation in this study enables us to examine practical insights from a rare leadership cohort.

The limitations of this study include the small sample size. The small sample size reflects the limited number of psychiatrist-CEOs currently practicing in the UK, as five eligible participants. The limited number of psychiatrist-CEOs also has the risk of potential identifiability of participants. As this study was carried out in the UK, it is possible that there is limited transferability in comparison to other countries. As this study is based on a self-reported questionnaire, there is also the potential for bias from the participants. 

With the aim of reducing author bias, the questionnaire feedback was anonymous, with the authors also being blind to participant responses. The questionnaire was designed with a combination of open and closed questions, with the option for the participant to include any further information they wanted to add in order to encourage open and honest feedback. All authors analysed the feedback in order to minimise the influence of a single author’s views. 

Future research should examine how psychiatrist-led organisations perform compared to others, particularly in mental health service delivery. 

## Conclusions

This study highlights how consultant psychiatrists' unique skills, including patient-centred thinking, crisis management, and emotional intelligence, make them suitable for CEO roles in healthcare. While not all psychiatrists may choose this path, support should be available for psychiatrists with an interest in leadership. Healthcare systems should provide bespoke leadership training, mentorship programs, and clear career pathways and address challenges like administrative burdens and work-life balance to sustain physician leaders in such demanding roles. By nurturing psychiatrist leadership, healthcare systems can leverage their clinical expertise at the executive level to drive meaningful improvements in care quality and organisational culture.

Future research should expand upon this work by seeking larger, more diverse international multi-country purposive sampling and adopting longitudinal qualitative panels, mixed-methods linking leader background to organisational metrics (staff turnover, serious incident rates, waiting times, and patient experience) to better understand the long-term impacts of psychiatric leadership on organisational and patient outcomes.

## Appendices

Study questionnaire

1.     How many years have you been practicing as a psychiatrist?

·      11-20 years

·      More than 20 years

2.     How many years have you been serving as the CEO of a mental health trust?

·      Less than 1 year

·      1-3 years

·      4-7 years

·      More than 7 years

3. Which aspects of your psychiatric training do you find most beneficial in your role as a CEO? Select all that apply.

·      Communication skills

·      Empathy and understanding

·      Crisis management

·      Analytical thinking

·      Patient-centred approach

·      Ethical decision-making

4. Can you describe a specific instance where your background as a psychiatrist directly influenced a decision you made as a CEO?

5. What are the main challenges you face in balancing your responsibilities as a CEO? Select all that apply.

·      Time management

·      Emotional burnout

·      Role conflict

·      Administrative burden

·      Maintaining clinical skills

·      Staff expectations

6. What advantages do you believe you have as a CEO because of your background in psychiatry?

7. What disadvantages or limitations have you encountered if you have a dual role as a psychiatrist and a CEO?

8. Do you have any advice for psychiatrists who aspire to take on leadership roles within mental health trusts?

9. Would you recommend other psychiatrists to pursue leadership roles such as CEO?

·      Strongly recommend

·      Recommend

·      Neutral

·      Do not recommend

·      Strongly do not recommend

10. Is there anything else you would like to share about your experience as a psychiatrist and CEO?
